# Nutraceuticals in Brown Adipose Tissue Activation

**DOI:** 10.3390/cells11243996

**Published:** 2022-12-10

**Authors:** Andrea Armani, Alessandra Feraco, Elisabetta Camajani, Stefania Gorini, Mauro Lombardo, Massimiliano Caprio

**Affiliations:** 1 Department of Human Sciences and Promotion of the Quality of Life, San Raffaele Roma Open University, 00166 Rome, Italy; 2 Laboratory of Cardiovascular Endocrinology, Istituto di Ricovero e Cura a Carattere Scientifico (IRCCS) San Raffaele, 00166 Rome, Italy; 3 Department of Experimental Medicine, Sapienza University of Rome, 00161 Rome, Italy

**Keywords:** brown adipocyte, obesity, thermogenesis, metabolic syndrome, microbiota, phytochemicals

## Abstract

Obesity and its associated comorbidities have become pandemic, and challenge the global healthcare system. Lifestyle changes, nutritional interventions and phamaceuticals should be differently combined in a personalized strategy to tackle such a public health burden. Altered brown adipose tissue (BAT) function contributes to the pathophysiology of obesity and glucose metabolism dysfunctions. BAT thermogenic activity burns glucose and fatty acids to produce heat through uncoupled respiration, and can dissipate the excessive calorie intake, reduce glycemia and circulate fatty acids released from white adipose tissue. Thus, BAT activity is expected to contribute to whole body energy homeostasis and protect against obesity, diabetes and alterations in lipid profile. To date, pharmacological therapies aimed at activating brown fat have failed in clinical trials, due to cardiovascular side effects or scarce efficacy. On the other hand, several studies have identified plant-derived chemical compounds capable of stimulating BAT thermogenesis in animal models, suggesting the translational applications of dietary supplements to fight adipose tissue dysfunctions. This review describes several nutraceuticals with thermogenic properties and provides indications, at a molecular level, of the regulation of the adipocyte thermogenesis by the mentioned phytochemicals.

## 1. Introduction

During the last decades, the prevalence of obesity has risen significantly worldwide. Obesity is associated with hormonal dysfunctions and systemic inflammation which contribute to insulin resistance, dyslipidemia, hypertension and metabolic syndrome (MetS), resulting in increased cardiovascular risk [1]. Anti-obesity pharmacological approaches currently either reduce absorption of dietary fat or decrease appetite [2]. So far, scarce long-term efficacy and potential adverse effects limit the use of pharmaceutical anti-obesity drugs [3], and phytochemicals may represent attractive options in term of negligible side effects and costs [4]. Two distinct types of fat are found in mammals, described as white adipose tissue (WAT) and brown adipose tissue (BAT). WAT is mainly deputed to store energy as triglycerides, whereas BAT dissipates energy as heat [5]. The presence of BAT is observed in rodents throughout life. In humans, albeit present in newborns and young children, BAT activity has historically been considered non relevant in adult subjects [6]. 

Studies performed in the early 2000s led to the discovery of metabolically active BAT in adult humans, thus providing a potential novel target to enhance energy expenditure (EE) and counteract obesity [7]. BAT is able to release energy in the form of heat, through the activity of the brown adipocyte-specific uncoupling protein 1 (UCP1), which generates heat by dissipating the proton gradient across the inner membrane of the mitochondria, with subsequent inhibition of ATP synthesis [7]. Such thermogenic activity, termed as non-shivering thermogenesis, contributes to regulating body temperature and can dissipate excess calories. Cold exposure leads to increased activity of sympathetic nervous system (SNS) fibers innervating BAT and represents a crucial mechanism of activation for BAT, promoting its thermogenic function [8]. Interestingly, cold stimulation not only activates BAT depots but also induces the emergence of brown-like adipocytes, termed “beige” or “brite” adipocytes, in WAT depots [9].

Such a process, called the “browning of WAT”, induces the formation of beige adipocytes, which reveal morphological and thermogenic properties similar to those of classical brown adipocytes [10]. Both preclinical and clinical studies have shown that BAT activation and/or browning of WAT are accompanied by increased EE and protection against overweight and glucose metabolism dysfunctions, indicating that stimulation of brown/brite adipocyte function results in beneficial effects on the metabolic health [9]. Cold-induced activation of BAT has been shown to improve insulin sensitivity both in healthy and in diabetic subjects [11,12]. 

Interestingly, it is debatable whether the raising of BAT activity and/or the induction of browning, which have been detected in murine tumor models and in oncological patients, may promote cancer-associated cachexia or, on the contrary, may curb tumor growth [13,14,15,16,17].

Pharmacological approaches aimed to stimulate brown fat activity in humans could be a potential strategy to target obesity and associated metabolic diseases [18]. BAT activation can be finely modulated by important endocrine axes with relevant implications on cardiometabolic health [19,20]. On the other hand, accumulating evidence reveals that brown and beige adipocyte thermogenic activity is also modulated by diet [4], suggesting that diets with different compositions in macronutrients may modulate brown fat activity differently [21]. Importantly, a number of studies over the last two decades have identified several nutritional compounds capable of stimulating the thermogenic function of brown and beige adipocytes [4]. This review aims to discuss a variety of nutraceuticals with documented abilities to boost the thermogenic activity of adipose tissue, also providing hints about molecular mechanisms by which such compounds modulate adipose tissue thermogenesis.

## 2. Molecular Pathways in Brown/Beige Fat Formation and Function

**Figure 1:**
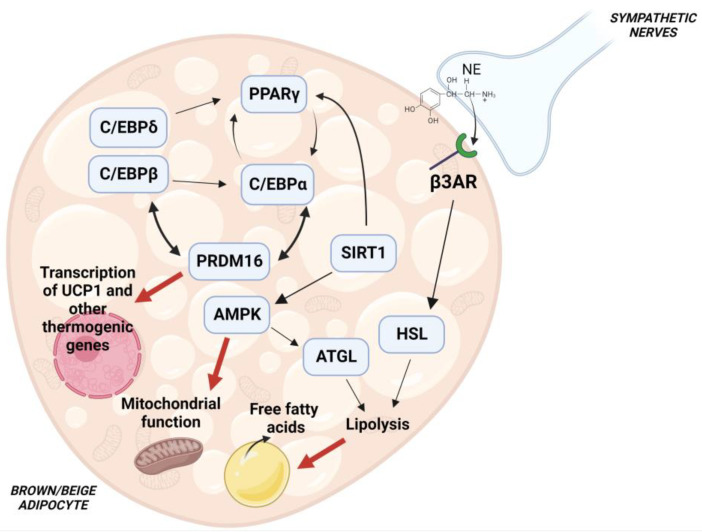
Molecular pathways regulating brown/beige adipocyte differentiation. Cooperation of PPARγ with C/EBPs is crucial for an optimal differentiation. PRDM16 promotes a thermogenic program thorugh interaction with C/EBPs and PPARγ, and loss of PRDM16 results in defective BAT formation. SIRT1 has been shown to deacetylate PPARγ. SIRT1-mediated deacetylation of PPARγ stimulates its interaction with PRDM16, leading to increased expression of brown and beige fat-specific genes. SIRT1 has also been shown to activate AMPK, a key enzyme involved in energy homeostasis. Activation of AMPK contributes to mitochodrial function by regulating the process of mitophagy, and stimulates ATGL activity to increase lipolysis. β-adrenergic receptor activation also stimulates lipolysis, with subsequent release of free fatty acids which promote mitochondrial β-oxidation and direct activation of UCP1.

Analysis of molecular mechanisms and signaling pathaways regulating white and brown adipocyte differentiation became an area of intense research activity in the early 1980s [22,23]. The current knowledge on this topic reveals that a number of transcription factors, transcriptional coactivators and corepressors form a complex transcriptional network which, in response to extracellular stimuli, regulates adipocyte differentiation [24]. A preminent role is played by the interaction of the transcriptional factors PPARγ with CCAAT-enhancer-binding proteins (C/EBPs), which directs both brown and white adipocyte differentiation [25]. Crucial contribution to brown and beige adipocyte differentiation is given by PRD1-BF-1-RIZ1 homologous domain containing protein 16 (PRDM16), which interacts with PPARγ and C/EBPs through its zinc finger motifs (ZF1, ZF2) [26] (Figure 1). SIRT1-dependent PPARγ deacetylation has been shown to promote its binding to PRDM16 with subsequent induction of thermogenic genes [27]. The protein deacetylase SIRT1 has also been found to promote the function of the AMP-activated protein kinase (AMPK), through deacetylation of the serine-threonine protein kinase LKB1 [28], and stimulate (see below) brown/beige fat formation. Activation of PPARγ as well as of PPARα, whose expression is higher in BAT than in WAT, increases expression of UCP1, PRDM16 and the PPARγ coactivator 1α (PGC-1α) which promotes mitochondrial biogenesis and β-oxidation [29,30]. Additional transciptional factors such as Krüppel-like factor 11, early B-cell factor and the EWS/YBX1/BMP7 axis have been found to affect PPARγ function and stimulate the expression of brown and beige fat genes, as described in detail elsewhere [29,31]. A key role in the regulation of the adipocyte thermogenic program is played by AMPK, a sensor of cellular energy status, which phosphorylates transcription factors and proteins involved in the formation and maintenance of brown and beige adipose tissue [32] (Figure 1).

Genetic removal of AMPK leads to the impairment in BAT formation [33] and WAT browning [34]. At a molecular level, AMPK has been found to modulate mitophagy and preserve mitochondrial function in the adipocyte [34]. In addition, AMPK has been shown to activate desnutrin/ATGL which hydrolyzes triacylglycerols to release fatty acids which, in turn, act as substrates for thermogenesis [35].

Cold exposure results in sympathetic activation of the thermogenic functions of brown and beige fat [36]. Norepinephrine (NE) is released from sympathetic nerve endings and binds to the adipocyte β3-adrenergic receptor, with a subsequent increase in cAMP and activation of protein kinase A (PKA) which promotes triglyceride hydrolysis [37,38]. Free fatty acids released by lipolysis both serve as fuel for mitochondrial β-oxidation and promote direct activation of UCP1 [39] (Figure 1). Exposure to cold temperature also stimulates AMPK function, leading to thermogenic effects [34]. Interestingly, the activation of molecular signaling pathways involved in brown/beige adipose tissue formation by nutraceutical compounds may represent a novel strategy to counteract obesity and its associated comorbidities [40].

## 3. Transient Receptor Potential Channels Activators

Transient receptor potential (TRP) channels represent a family of non-selective ion channels, found in several cell types, including sensory neurons and adipocytes [4]. A number of studies has shown that TRP channels regulate thermoregulation by stimulation of the SNS, with subsequent BAT activation and increased EE. Several dietary compounds have been found to activate TRP channels [4]. Capsaicin and capsinoids are present in chili peppers and act as potent activators of the TRP vanilloid 1 (TRPV1), a channel which exhibits high permeability to calcium and can be activated by toxins and temperatures higher than 42 °C, revealing a role as a receptor of noxius stimuli [41]. Preclinical data by Kawabata et al. [42] show that stimulation of gastrointestinal TRPV1 by capsinoids promotes BAT activity and EE through activation of the extrinsic nerves in the gastrointestinal tract. Gastrointestinal vagal afferents project to nucleus tractus solitarii, which regulates the sympathetic nervous stimulation to BAT [43], thus suggesting that brown adipocyte activation is mediated by TRPV1 through the stimulation of sympathetic nerves. 

On the other hand, TRPV1 expression has been detected in WAT and BAT [44], indicating potential cell-autonomous effects of TRPV1 activation in brown adipocytes, thus leading to increased BAT thermogenesis through local activation of TRPV1. Treatment of brown adipocytes with capsaicin was able to promote calcium influx and increase the expression of brown adipogenesis markers such as PPARγ and PGC-1α [45]. Of note, the treatment of differentiating 3T3-L1 preadipocytes with capsaicin was found to promote the expression of several thermogenic genes known to be upregulated during the process of “browning” (i.e., PGC-1α, NCOA1, FOXC2, PRDM16, SIRT1), suggesting that stimulation of adipose-specific TRPV1 may induce the “beige” phenotype [46]. In accordance with these findings, treatment of mice with a capsaicin analog (capsiate) resulted in increased levels of UCP1 in BAT with a parallel rise in metabolic rate [47]. The involvement of TRPV1 was confirmed by studying TRPV1KO mice treated with capsaicin that were not protected from diet-induced obesity [48]. In humans, intake of capsinoids results in enhancement of EE and BAT activation, also revealing anti-obesogenic effects, confirming the preclinical data and suggesting that intake of foods containing capsinoids may represent an efficient strategy to reduce fat mass accumulation [42,49]. At a molecular level, in the adipocyte, the opening of TRPV1 promotes Ca^2+^ influx which may activate the Ca^2+^/Calmodulin-stimulated protein kinase kinase β and, in turn, AMPK, which is known to stimulate the thermogenic gene program through the increased expression of PRDM16 [32,50,51].

As shown by cell-based experiments, other food compounds such as the black pepper components piperine, isopiperine, isochavicine, piperanine, piperolein A and B, [52]; sulfides present in garlic such as diallyl sulfide, diallyl disulfide and diallyl trisulfide [53]; and gingerols and shogaols occuring in ginger [54,55], are able to activate TRPV1 as well as TRP ankyrin 1 (TRPA1), another member of the TRP family. In rats fed a high fat diet (HFD), allyl-containing sulfides of garlic may mediate the increase of UCP1 and the anti-obesity effects of garlic oil administration [56]. In humans, intake of grains of paradise or black ginger extract has been shown to increase EE through BAT activation [57,58,59]. Ginger supplementation in mice fed a HFD counteracted fat accumulation through stimulation of BAT function and activation of WAT browning [59]. In addition, in mice, dietary supplementation with fish oil rich in fatty acids eicosapentaenoic acid and docosahexaenoic acid has been shown to activate TRPV1 and increase UCP1 expression in brown/beige adipocytes through stimulation of SNS, suggesting that omega-3 fatty acids are potential activators of thermogenic fat [60], potentially countearacting fat mass expansion via TRPV1 activation. Oleuropein aglycone, a polyphenol abundant in extra virgin olive oil, and allyl isothiocyanate, which can be found in yellow mustard, wasabi and menthol, act as agonists of TRPA1, whose activation stimulates β-adrenergic signaling and promotes brown fat thermogenic activity in rodents [61]. These findings suggest that also dietary TRPA1 ligands may be an efficient approach to treat obesity.

In addition, menthol represents a ligand for the TRP melastatin 8 (TRPM8) channel, a TRP family member whose activation increses UCP1 expression in adipocyte cultures. Such effects of menthol were observed also in the adipose tissue of mice [62]. Expression of TRPM8 in brown adipocytes suggests that TRPM8 ligands can directly activate BAT. Importantly, TRPM8-induced BAT activation was able to prevent obesity and glucose intolerance in mice fed a HFD [63]. Interestingly, genetic polymorphisms identified in the TRPM8 gene have been associated with different suscetibility to MetS, further suggesting the TRPM8 function, and its modulation by dietary ligands, may affect adipose tissue and glucose metabolism [64].

## 4. Green Tea Compounds (Catechins and Caffein)

A number of studies have shown that intake of green tea catechins, i.e., the polyphenols epigallocatechin gallate (EGCG) and epigallocatechin, stimulates thermogenic fat activity [4]. In rats fed an HFD, supplementation with catechins leads to reduced white fat mass and increased expression of UCP1 in BAT, suggesting anti-obesogenic effects of these polyphenolic substances [65]. In accordance with this data, mice treated with theaflavins showed an increase in EE, with a concomitant increase of UCP1 and PGC-1α levels in BAT [66]. In mice fed an HFD, green tea extract supplemention was able to increase EE as well as protein content of BAT, and dampen weight gain [67]. These data hint that induction of BAT thermogenesis could mediate the effects of catechin administration. Cotreatment of mice with the β-adrenoceptor antagonist propranolol prevented such effects, indicating a crucial role for the adrenergic pathway in eliciting the anti-obesity actions of tea catechins [67]. Mechanistically, catechins were found to inhibit catechol-O-methyltransferase, an enzyme involved in the degradation of NE, and this enzymatic activity is expected to promote the sympathetic stimulation of BAT [68]. Induction of browning was also observed in the WAT of obese rats treated with green tea extract. The WAT of these rodents revealed upregulated expression of genes involved in beige adipocyte formation such as PPARγ, PRDM16 and bone morphogenetic protein-7 (BMP-7) [69].

The alkaloid caffein is present in green tea extracts, and can promote lipolysis and exert thermogenic function in adipocytes through the local rise in cAMP intracellular levels [70]. Mice treated with caffeine displayed the increased thermogenic activity of BAT [71] and, accordingly, additional experiments showed that exposure to caffeine raised levels of UCP1 and PGC-1α, and promoted mitochondrial biogenesis in adipocyte cultures [72]. Enhancement of UCP1 function by caffein has been suggested to be mediated by inhibition of adipocyte phosphodiesterase, with subsequent increases in cAMP levels and PKA activation which, in turn, stimulates UCP1 activity [73,74].

Enhancement of human BAT activity, as indicated by fluorodeoxyglucose-positron emission tomography, has been shown after oral ingestion of a green tea extract (50 mg caffeine and 90 mg EGCG), with a parallel increase in EE [75]. This study, as previously observed by Dulloo et al. [76], suggested a more decisive impact of EGCG, rather than of caffeine, on EE. On the other hand, drinking coffee has been shown to raise the superclavicular temperature, i.e., in a region which colocates with brown fat, and suggested t caffeine efficiently promotes the activation of BAT in humans [72]. These conflicting results in the response to caffein may be explained by differences in doses and duration of the studies. Several studies have investigated if the thermogenic activity induced by catechins and caffein is able to counteract fat expansion and body weight gain in humans, and a meta-analysis performed by Phung et al. shows that intake of catechins and caffein, compared with caffein alone, is more efficient in reducing BMI, body weight and waist circumference, suggesting that catechins and caffein synergize in regulating body fat mass [77,78,79,80].

## 5. Flavonoids

In addition to the abovementioned flavanols, epigallocatechin and EGCG, other subfamilies of flavonoids have been investigated as modulators of brown adipocyte thermogenic function. Flavonoids are present in fruits, vegetables, tea and wine and are associated with protection against type 2 diabetes, obesity and cadiovascular disease [81]. A favourable impact of the flavanone hesperidin has been observed on lipid profiles and blood pressure [82]. Treatment of rats with G-hesperidin (4G-alpha-glucopyranosyl hesperidin), a glucosyl derivative of hesperidin which is more water-soluble and efficiently absorbed, shows the increased activity of sympathetic nerves innervating BAT with potential thermogenic effects [82]. A study performed on mice treated with α-monoglucosyl hesperidin (αGH), another derivative of hesperidine, revealed a reduction in white fat depots that was mediated by the induction of brown-like adipocyte formation, indicating that αGH-induced browning of inguinal WAT could mediate the observed increase in thermogenesis which led to decreased body fat deposition [83]. At a molecular level, αGH has been suggested to act as an agonist of PPARγ which, in turn, stabilizes PRDM16 which promotes a brown fat-specific gene program [26,83] (Figure 1).

As observed by cell culture-based experiments, treatment of 3T3-L1 murine adipocytes with a concentrated water extract of *Prunus mume* fruit, rich in naringin, was able to promote expression of genes involved in mitochondrial biogenesis genes, (NAMPT, Nrf1, Nrf2, CPT1α) and in brown-like adipocyte differentiation (PGC-1α, UCP1, CIDEA, Cox7α1, Cox8b) also reducing, in parallel, reactive oxygen species abundance. These data suggested the flavanone naringin is able to favour the conversion of white to brite adipocytes, potentially representing a coumpound suitable for counteracting obesity development [84] and, interestingly, a clinical study revealed that treatment with the flavonoids naringin or hesperidin in combination with the alkaloid p-synephrine (see below) enhanced the effects of p-synephrine to increase the resting metabolic rate of the participants [85].

Quercetin is abundant in berries, apples, red onions, grapes, broccoli, and other vegetables, and preclinical studies have shown that such a flavonol stimulates browning of WAT. In mice, dietary supplementation with quercetin increased UCP1 expression in WAT and BAT, thus promoting browning of WAT and BAT activity [86]. Quercetin treatment resulted in the rise of plasma NE levels, stimulating activation of cAMP-dependent PKA and AMPK, two crucial players for brown/beige adipocyte function [87]. Stimulation of βARs in brown and white adipocytes has been shown to raise levels of intracellular cAMP, with subsequent activation of PKA and AMPK [88], as observed upon treatment with quercetin which, in fact, resulted in the sympathetic activation of thermogenic adipose tissue. Of note, quercetin was also able to increase the abundance of PPARγ and PGC1α proteins, which could further stimulate UCP1 expression and mitochondrial biogenesis in the adipose tissue [86]. Accordingly, quercetin was able to induce browning of WAT in obese mice, and to improve glucose and lipid metabolism, thus suggesting anti-obesity effects [89].

A number of studies have shown that flavones are compounds capable of regulating lipid and glucose profiles, counteracting inflammation and oxidative stress [90]. Thermogenic properties of luteolin have been investigated in mice fed a HFD supplemented with such flavones [91]. Dietary luteolin was able to prevent HFD-induced body weight gain, fat expansion and glucose metabolism alterations, and increase EE. Such metabolic effects were associated with stimulation of the thermogenic gene program both in WAT and in BAT. Luteolin treatment mechanistically increased protein abundance of AMPK, SIRT1 and PGC-1α, which together form a molecular network modulating EE [92]. Of note, the cotreatment of luteolin-treated primary brown and subcutaneous adipocytes with an AMPK inhibitor repressed the increase of SIRT1, PGC-1α and thermogenic genes, confirming the pivotal role of AMPK in mediating the effects of luteolin [91].

Isoflavones, also called phytoestrogens, represent another category of flavonoids which show beneficial effects on type 2 diabetes and obesity in preclinical studies. Soy isoflavones, such as genistein and daidzein, have been shown to counteract hepatic steatosis in obese rats or mice through reduction in lipogenesis and increased fatty acid oxidation [93]. In the adipose tissue, soy isoflavones promote the activation of AMPK and ATGL, with a parallel reduction of SREBP1 protein levels, resulting in reduced lipid accumulation and fat mass expansion [94]. In addition to the inhibition of fat accumulation, the induction of browning has been observed in adipocyte cultures treated with genistein showing reduced expression of genes enriched in white adipocytes, and displaying increased levels of of brown/beige adipocyte-specific transcipts, including UCP1. Interestingly, cotreatment with an inhibitor of SIRT1 repressed the rise in UCP1 levels, indicating the involvement of SIRT1 in mediating the formation of beige adipocyte induced by genistein [95]. Such effects were confirmed also in vivo, in obese mice with dietary supplementation of genistein which displayed reduced obesity and improved glucose metabolism and the induction of WAT browning [96].

Interestingly, another study showed that rats treated with genistein displayed improved adiposity and insulin sensitivity associated with increased plasma levels of the myokine irisin [97,98]. Released from skeletal muscle, irisin promotes WAT browning which is expected to protect against dysregulated fat mass expansion and glucose metabolism alterations [99]. The induction of browning through enhancement of irisin shows an additonal mechanism by which genistein may stimulate beige adipocyte formation. Similar metabolic responses, in terms of reduced expression of lipogenesis with concomitant stimulation of browning, were also observed in mice exposed to the isoflavone daidzein which resulted in reduced fat mass and decreased protein abundance of the lipogenic enzyme stearoyl coenzyme A desaturase 1, paralleled by an increase in UCP1 protein levels [100]. To date, there is no evidence that dietary supplementation with isoflavones also leads to the mentioned metabolic effects on adipose tissue in humans [101].

## 6. Stilbenes

Resveratrol and pterostilbene (PTS) are two polyphenols belonging to the category of the stilbenes, present in grapes and blueberries, which show the ability to modulate molecular pathways regulating cell senescence, as observed in a number of preclinical studies [102]. Anti-oxidant and anti-inflammatory effects have been diplayed by resveratrol and PTS [102] which are also able to regulate the thermogenic capacity of adipose tissue, at least in experimental models [103]. Mice fed a HFD supplementated with resveratrol showed reduced weight gain, which was associated with an increased formation of brown adipocytes in the interscapular brown fat depots. Dietary resveratrol led to an increased expression of brown adipogenic markers, including PRDM16 and UCP1, via enhancement of adipocyte-specific AMPK activity [104]. Another study perfomed on HFD mice treated with resveratrol showed the induction of brown-like adipocyte formation in inguinal WAT (iWAT), confirming the involvement of AMPK in mediating the effects of resveratrol [105]. Both these studies observed brown and beige adipocyte formation in adipocyte cultures from either iBAT or iWAT upon ex vivo treatment with resveratrol, showing also cell-autonomous effects of this stilbenoid. Interestingly, 3T3-L1 cells treated with resveratrol showed increased expression of beige adipocyte markers through activation of mammalian target of rapamycin (mTOR), suggesting the involvement of this serine/threonine kinase as an additional mediator of resveratrol [106], even though the role of mTOR in the regulation of beige adipocyte formation is still controversial [107].

In addition, a study by Lee et al. showed that the NAD^+^-dependent deacetylase SIRT1 mediates the protective effects of resveratrol observed on glucose and lipid profiles in mice, including the induction of browning [108]. At the molecular level, SIRT1 induces “browning” through deacetylation of PPARγ on Lys268 and Lys293. Deacetylation of these two residues allows the recruitment of PRDM16 to PPARγ, with subsequent stimulation of the thermogenic gene program in the adipose tissue [27]. AMPK has been proposed to induce resveratrol-induced SIRT1 activation by increasing NAD^+^ levels [109] through enhancement of the the NAD+ biosynthetic enzyme nicotinamide phosphoribosyltransferase (NAMPT) [110]. There are several preclinical and clinical studies showing that resveratrol administration results in anti-obesity effects [111]. In particular, in MetS patients treated with resveratrol, there was a reduction in weight, BMI, fat mass, waist circumference and insulin levels [112]. A meta-analysis performed by Tabrizi et al. has shown that resveratrol administration reduces weight, fat mass and BMI [113], suggesting that such effects may be mediated by BAT enhancement, even though the modulation of adipose tissue thermogenic function has not been observed in humans. In subjects with type 2 diabetes, resveratrol supplementation has been shown to improve ex vivo mitochondrial function but did not affect BAT activity [114].

Reduced bioavailability of resveratrol may limit its biological activity and beneficial effects on adipose tissue [103], and several studies focused on pterostilbene, a natural dimethylated analog of resveratrol which has displayed improved pharmacokinetic properties [115]. Pterostilbene has been found to induce expression of thermogenic genes in white adipocyte cultures as well as in WAT of mice [116]. Another study has observed that dietary supplementation with pterostilbene resulted in reduction in fat mass, with a parallel higher expression level of thermogenic and oxidative genes in iBAT of rats [117]. Increased expression of PPARα, induced by pterostilbene, was suggested to contribute to the rise in UCP1 levels [118]. However, neither anti-obesity effects nor induction of thermogenic adipocytes have been reported in clinical studies, suggesting that further investigations are required to study the efficacy of pterostilbene on human adipose tissue [119].

## 7. Sympathomimetics and Other Thermogenic Compounds

In addition to caffein, ephedrine and synephrine have putative sympathomimetic activity by stimulating catecholamine release from sympathetic nerves [4]. A study peformed in 1984 suggested that treatment of mice with ephedrine, extracted from *Ephedra* plants, was able to increase the abundance of a specific 32,000 molecular weight, GDP-binding protein in the mitochondria, and these data were considered as an indication of the increased thermogenic activity of BAT [120]. More recent evidence shows that the acute oral intake of ephedrine results in increased BAT activity, measured by using (18)F-fluorodeoxyglucose positron emission tomography-computed tomography (FDG-PET) in lean, but not in obese, subjects [121]. In another study, long-term ephedrine treatment led to reduction in body fat but did not stimulate BAT activity, excluding that the decrease in WAT mass could derive from BAT-mediated adaptive thermogenesis and that chronic treatment with ephedrine can enhance BAT function [122]. These findings suggest that the metabolic profile or duration of the treatment may affect the outcome in humans. On the other hand, obese mice treated with ephedrine displayed reductions in body weight and fat, with a parallel rise in oxygen consumption and an increased expression of mitochondrial biogenesis-related genes and UCP1 in BAT [123], thus showing a robust response in terms of fat brown activation, in contrast with the previous mentioned clinical study. These data suggest a higher responsiveness to ephedrine for the murine BAT, compared with humans.

Synephrine is a sympathomimetic amine, found in *Citrus aurantium,* whose administration has been shown to increase oxygen consumption, EE and lipid oxidation in healthy men [124]. Interestingly, other studies showed that synephrine intake resulted in increased EE and fat oxidation rate during physical exercise [125,126]. Treatment of mouse inguinal preadipocyte cultures with p-synephrine has been shown to induce expression of brown/beige adipocyte markers, including UCP1 [127]. Of note, UCP1 induction was repressed by cotreatement with a β3-adrenoceptor-specific antagonist, revealing the potential ability of p-synephrine to bind and activate adipocyte β3-adrenoceptor, with subsequent direct stimulatory effects on thermogenic adipocyte differentiation [127]. However, BAT function modulation by supplementation with synephrine has not been observed in clinical studies.

Over the last years, a number of studies have shown that berberin exerts hypolipidemic effects through increased expression of hepatic LDL receptor, suggesting a potential therapeutic use of this alkaloid to treat metabolic diseases such as obesity and diabetes [128]. Cell culture-based experiments and animal studies have revealed that berberin also represses white adipogenesis, via the up-regulation of C/EBP inhibitors such as CHOP and DEC2 [129], and promotes BAT activity and browning of WAT in obese mice. Mechanistically, such effects on adipose tissue were mediated by local activation of AMPK with, in turn, up-regulation of PGC-1α and increased levels of UCP1 [130]. Berberine has also been shown to repress white adipocyte proliferation and differentiation through down-regulation of galectin-3, a protein which contains a carbohydrate-recognition binding domain and affects expression and transcriptional activity of PPARγ [131,132]. In addition, berberine was able to stimulate brown adipogenesis in cultures of human adipocytes, via increased demethylation of the PRDM16 promoter and subsequent increased transcription of this master regulator of brown/beige adipogenesis [133] (Figure 1). A systematic review and meta-analysis of human studies on berberine supplementation has observed that intake of this alkaloid led to a decrease in body weight and reduction in BMI and waist circumference, indicating the anti-obesity effects of berberine [134]. Other meta-analyses showed the beneficial effects of berberine intake on blood glucose metabolism in type 2 diabetes patients [135] as well as improvement of the lipid profile in terms of reduced levels of total cholesterol, triglycerides and LDL cholesterol [136]. If such beneficial effects of berberin in clinical trials could be ascribed, at least in part, to its ability to promote brown fat function, is an issue which still needs to be addressed and requires further studies.

Vitamin A is known to be involved in cell proliferation and differentiation, immunity, reproduction and retinal function [137]. The participation of vitamin A in several crucial physiological processes can be explained by considering that retinoic acid and its metabolites act both as transcriptional regulators and modulators of extranuclear signaling transduction cascades in different cell types [138]. Vitamin A and its metabolites regulate gene expression by modulating transcriptional activity of the retinoic acid receptors RAR, RXR and PPAR [137]. Retinoic acid-responsive elements have been found in the regulatory regions of UCP1 gene, and incubation of brown adipocyte cultures with retinoic acid increased UCP1 transcript levels [139]. Transcriptional regulation of UCP1 by retinoids is mediated mainly by RARα, RARβ and RXRα [140], even though retinoic acid has also been shown to activate p38 mitogen-activated protein kinase which, in turn, promotes transcription of UCP1 [141,142]. On the other side, in 3T3-L1 cultures, retinoic acid was found to reduce the expression the lipogenic transcription factors and the intracellular lipid content [143]. Interestingly, the retinoic acid precursor retinaldehyde (Rald) has been found in mouse and human fat depots, and studies in adipocyte cultures showed that Rald treatment stimulated the expression of UCP1 through the recruitment of RARα and PGC-1α to the promoter of UCP1 [144]. Of note, treatment of obese mice with Rald was able to repress fat mass expansion [145], further suggesting that retinoids are able to modulate adipose tissue metabolism. Clinical studies on the potential relationship between retinoids and activation of thermogenic fat are not available, and clinical trials are required to investigate such effects in humans.

## 8. Nutraceuticals and Microbiota

The composition of the gut microbiota has been shown to affect host metabolisms and energy homeostasis, and regulate the thermogenic function of brown fat [146]. The altered gut microbiota profile has been recognised to contribute to the pathogenesis of metabolic alterations such as obesity, diabetes and MetS [147]. In particular, an increased ratio of Firmicutes to Bacteroidetes, observed in obese mice, has been suggested to contribute to obesity development [148]. Such a profile has been found in the gut microbiota of obese humans, compared with lean subjects, with the abundance of Bacteroidetes in obese subjects increasing after weight loss [149]. Reduction in gut microbiota richness has also been proposed to contribute to the pathophysiology of obesity [150].

Bacterial genera such as Bacteroides, Faecalibacterium, and Clostridium are able to produce and modulate total levels and relative proportions of specific short-chain fatty acids (SCFAs) in the systemic circulation [151,152]. Several genera in the phylum *Bacteroidetes* produce acetate and propionate, whereas butyrate mostly derives from genera in the phylum *Firmicutes* [153]. Circulating SCFAs have been proposed to regulate a variety of metabolic processes in different organs and tissue, at least in animal models, including adipose tissue thermogenic function [151,152]. Treatment of brown or white adipocyte cultures with SCFA, i.e., acetate, has shown the induction of brown fat markers [151] and, in mice, acetate or butyrate adminsitration was able to stimulate beige adipocyte differentiation [154] and BAT activation [155].

Preclinical studies have shown that nutraceuticals capable of modulating the microbiota profile can affect thermogenic fat activation. Obese mice treated with the flavonoid tangeretin displayed a reduction in body weight, liver steatosis and improved glucose metabolism [156]. Of note, tangeretin treatment was able to alter the gut microbiota composition and stimulate BAT activity, counteracting the dysbiosis and reducing the ratio of Firmicutes to Bacteroidetes [156]. In another study, mice fed a HFD supplemented with the natural polymethoxyflavone nobiletin showed reduced obesity and induction of WAT browning, along with a composition shift in the gut microbiota, which displayed an increase in the abundance of Bacteroidetes and in the ratio of Bacteroidetes to Firmicutes [157]. Interestingly, transplantation of the microbiota from mice treated with nobiletin to obese mice resulted in increased BAT activity, induction of beige adipocyte formation and reduced obesity in the recipient animals, indicating that nobiletin was capable of modifying the gut microbiota composition, leading to increased gut microbial production of acetate and subsequent enhancement of adipose tissue thermogenic activity [157]. Other phytochemicals such as quercetin and L-theanine were found to modulate the gut microbiota composition, increase the intestinal production of SCFAs and stimulate the thermogenic activity of adipose tissue in mice treated with either compound [156,158,159].

Transplantation experiments of fecal microbiota from mice treated with resveratrol to microbiota-depleted mice further revealed a causal relationship between specific changes in the gut microbial community composition and WAT browning [160]. The mentioned studies suggest that the gut microbiota–adipose tissue axis might account for the activating effects on adipose tissue thermogenesis by dietary phytochemicals. However, such evidence derives from preclinical studies, and clinical trials are deemed necessary to confirm the occurrence of this axis in humans.

## 9. Conclusions

**Figure 2:**
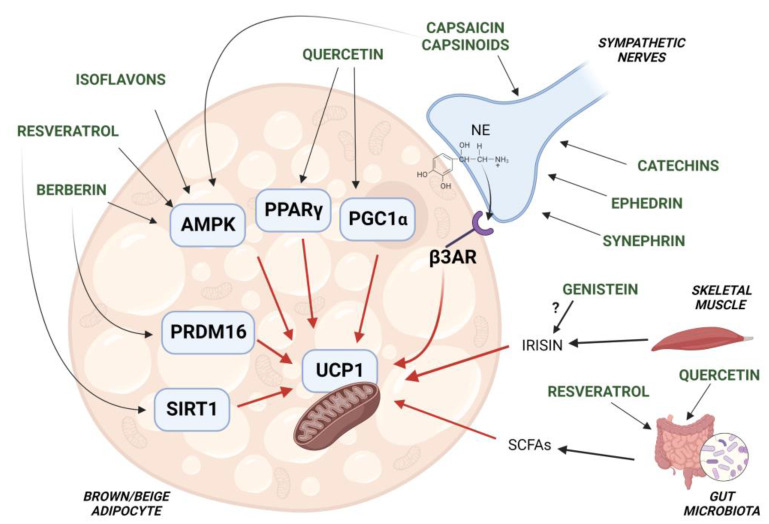
Schematic representation of the molecur targets involved in brown/beige adipocyte thermogenesis, which are regulated by nutraceuticals. UCP1 function of brown/beige adipocytes is activated by capsinoids, catechins, ephedrine and synephrin via stimulation of the sympathetic nervous system (SNS). Other phytochemicals such as quercetin, resveratrol, isoflavones and berberin directly promote the activation of factors (i.e., PPARγ, AMPK, PRDM16) which increase UCP1 function and expression. Adipocyte AMPK is also activated by capsinoids. Both resveratrol and quercetin have been shown to affect gut microbiota function, resulting in increased production of SCFAs which stimulate UCP1-dependent thermogenesis. Of note, the isoflavone genistein has been shown to increase the circulating levels of the myokine irisin which, in the adipose tissue, induces expression of thermogenic genes, including UCP1.

A number of studies suggest that the activation of BAT thermogenesis represents as a novel strategy to counteract obesity and associated metabolic diseases. The use of phrmacological agents, capable of activating human BAT, has found a limited success due to associated cardiovascular side effects [9]. As described above, specific nutraceuticals, provided also as combinations of different compounds, have been found to induce thermogenic gene expression in the adipose tissue of animal models. Preclinical studies have allowed the identification of molecular targets and signaling pathways involved in brown/beige adipocyte thermogenesis, which are modulated by dietary supplements. Up-regulated expression of PPARγ, C/EBPs, PGC-1α and PRDM16, with concomitant increases in the thermogenic protein UCP1, were found to be induced by nutraceuticals such as capsinoids, catechins, sympathomimetics and flavonoids [67,86,161,162] (Figure 2). Thermogenic activation of adipose tissue has been associated with reduction of white fat mass in studies with obese rodents treated with phytochemicals, supporting that such compounds can counteract excessive expansion of WAT through the activation of brown/beige adipocytes [163].

Interestingly, a number of studies have shown the activation of brown/beige adipose tissues in animal models of cancer [14,164,165,166] and in patients with different types of tumor (such as hepatocellular carcinoma, pancreatic adenocarcinoma, anaplastic carcinoma of the lung, etc.) [15,167,168,169]. Such thermogenic activation has been suggested to contribute to the increased EE in cancer-associated cachexia, a condition characterized by a remarkable body-weight reduction. However, the role played by BAT in cancer progression is still controversial. Recent evidence has shown that cold-activated BAT counteracts cancer growth in tumor-bearing mice [16]. Several types of tumor use glycolisis to produce energy for their development and progression [170], and the activation of thermogenic fat stimulates the uptake of blood glucose by adipocytes, with subsequent reduction in circulating levels of this fuel for cancer cells [16]. In addition, BAT has been proposed to release anti-inflammatory cytokines that could dampen tumor growth [17]. Under this perspective, nutraceutical supplements for BAT activation may be combined with cancer treatments to improve prognosis in oncological patients.

Notably, recent studies suggest that changes in the composition of gut microbiota affect the thermogenic function of BAT and WAT, contributing to EE and conferring protection against obesity and associated diseases [147]. There is evidence that specific changes in gut microbiota composition and metabolite production by nutraceuticals result in BAT activation and WAT browning [152]. Therefore, thermogenic fat activation by nutraceutical compounds can rely on different mechanisms, i.e., increased outflow of SNS to adipose tissue, enhancement of thermogenic function of adipocytes in a cell-autonomous manner or modulation of the gut microbiota metabolism (Figure 2).

Most of the aformentioned studies nevertheless provide data from experimental animal and/or cell-based models, and clinical trials are needed to investigate the effects on adipose tissue thermogenesis by nutraceuticals in humans. In clinical studies, it should be kept in mind that individual responsiveness to thermogenic compouds may potentially be influenced by the metabolic profile, in terms of degree of obesity, presence of different features of MetS and genetic predisposition to developing BAT. Nutraceuticals capable of stimulating BAT thermogenesis may thus be considered as a promising approach to be adopted in the context of programs of cardiometabolic rehabilitation.

Indeed, the use of nutritional compounds may provide significant advantages, in term of the costs and safety of therapeutic approaches, to counteract adipose tissue dysfunctions and associated metabolic disorders.

## Data Availability

Not applicable.
